# Ciprofloxacin-resistant Salmonella enterica Typhimurium and Choleraesuis from Pigs to Humans, Taiwan

**DOI:** 10.3201/eid1001.030171

**Published:** 2004-01

**Authors:** Po-Ren Hsueh, Lee-Jene Teng, Sung-Pin Tseng, Chao-Fu Chang, Jen-Hsien Wan, Jing-Jou Yan, Chun-Ming Lee, Yin-Ching Chuang, Wen-Kuei Huang, Dine Yang, Jainn-Ming Shyr, Kwok-Woon Yu, Li-Shin Wang, Jang-Jih Lu, Wen-Chien Ko, Jiunn-Jong Wu, Feng-Yee Chang, Yi-Chueh Yang, Yeu-Jun Lau, Yung-Ching Liu, Cheng-Yi Liu, Shen-Wu Ho, Kwen-Tay Luh

**Affiliations:** * National Taiwan University College of Medicine, Taipei, Taiwan; † The Study Group of Surveillance from Multicenter Antimicrobial Resistance in Taiwan, Taipei, Taiwan; ‡ Graduate Institute of Veterinary Medicine, National Taiwan University, Taipei, Taiwan

**Keywords:** ciprofloxacin resistance, Salmonella enterica Typhimurium, Salmonella enterica Choleraesuis, Taiwan

## Abstract

We evaluated the disk susceptibility data of 671 nontyphoid Salmonella isolates collected from different parts of Taiwan from March 2001 to August 2001 and 1,261 nontyphoid Salmonella isolates from the National Taiwan University Hospital from 1996 to 2001. Overall, ciprofloxacn resistance was found in 2.7% (18/671) of all nontyphoid Salmonella isolates, in 1.4% (5/347) of Salmonella enterica serotype Typhimurium and in 7.5% (8/107) in S. enterica serotype Choleraesuis nationwide. MICs of six newer fluoroquinolones were determined for the following isolates: 37 isolates of ciprofloxacin-resistant (human) S. enterica Typhimurium (N = 26) and Choleraesuis (N = 11), 10 isolates of ciprofloxacin-susceptible (MIC <1 μg/mL) (human) isolates of these two serotypes, and 15 swine isolates from S. enterica Choleraesuis (N = 13) and Typhmurium (N = 2) with reduced susceptibility to ciprofloxacin (MIC >0.12 μg/mL). Sequence analysis of the gryA, gyrB, parC, parE, and acrR genes, ciprofloxacin accumulation; and genotypes generated by pulsed-field gel electrophoresis with three restriction enzymes (SpeI, XbaI, and BlnI) were performed. All 26 S. enterica Typhimurium isolates from humans and pigs belonged to genotype I. For S. enterica Choleraesuis isolates, 91% (10/11) of human isolates and 54% (7/13) of swine isolates belonged to genotype B. These two genotypes isolates from humans all exhibited a high-level of resistance to ciprofloxacin (MIC 16–64 μg/mL). They had two-base substitutions in the gyrA gene at codons 83 (Ser83Phe) and 87 (Asp87Gly or Asp87Asn) and in the parC gene at codon 80 (Ser80Arg, Ser80Ile, or Ser84Lys). Our investigation documented that not only did these two S. enterica isolates have a high prevalence of ciprofloxacin resistance nationwide but also that some closely related ciprofloxacin-resistant strains are disseminated from pigs to humans.

Infections caused by nontyphoid Salmonella in humans are increasingly frequent in developed and developing countries (*1*,*2*). The increasing rates of resistance to traditional anti-Salmonella agents (i.e., ampicillin, chloramphenicol, and trimethoprim-sulfamethoxazole) and extended-spectrum cephalosporins among these isolates have made treatment of invasive salmonellosis a clinical dilemma (*3*–*6*). Of particular concern is the emergence of fluoroquinolone resistance among nontyphoid Salmonella and the occurrence of outbreaks caused by some resistant clones, since this class of antimicrobial agents constitutes the drug of choice for treating potentially life-threatening Salmonella infections caused by the multidrug-resistant strains in adult persons (*7*–*16*). Moreover, cases of treatment failure due to fluoroquinolone resistance in Salmonella strains have been reported (*17*–*19*).

Researchers have increasingly reported that widespread use of fluoroquinolones in food animals leads to the rapid emergence and dissemination of resistant Salmonella infections to humans, particularly in developing countries (*4*,*8*,*20*–*23*). In Taiwan, Chiu et al. reported that resistance to ciprofloxacin among S. enterica Choleraesuis isolates first appeared in 2000 and <60% of the isolates recovered from two hospitals in northern Taiwan in the third quarter of 2001 were resistant to ciprofloxacin (*14*). Molecular investigation clearly demonstrated that the primary sources of these resistant strains were herds of pigs.

To better understand the prevalence of nationwide resistance and the probable dissemination of ciprofloxacin-resistant nontyphoid Salmonella isolates, particularly S. enterica Typhimurium and Choleraesuis, we determined the mechanisms of quinolone resistance and the genotypes of ciprofloxacin-resistant isolates from humans and pigs, collected in different parts of Taiwan. This study is part of the Surveillance from Multicenter Antimicrobial Resistance in Taiwan (SMART) programs conducted in 2001.

## Materials and Methods

### Bacterial Isolates

A total of 671 nontyphoid Salmonella isolates were collected for the study. These isolates were recovered from various clinical specimens of patients treated at 11 major hospitals (bed capacities from 800 to 2,000) in different regions of Taiwan. These hospitals included the National Taiwan University Hospital (NTU; hospital A), Taipei; Taipei Veterans General Hospital (hospital B), Taipei; Mackay Memorial Hospital (hospital C), Taipei; Tri-service General Hospital (hospital D), Taipei; Taichung Veterans General Hospital (hospital E), Taichung; China Medical College Hospital (hospital F), Taichung; National Cheng-Kung University Hospital (hospital G), Tainan; Chi-Mei Medical Center (hospital H), Tainan; Kaohsiung Veterans General Hospital (hospital I), Kaohsiung; and Tzu-Chi General Hospital, Hualien (hospital J). Of the 671 isolates tested, 429 (64%) were recovered from stool samples, 141 (21%) from blood, and the rest from various body fluids.

Disk diffusion susceptibility results on these isolates were also provided by the hospitals and evaluated. Organisms were categorized as susceptible or resistant (including intermediate isolates) to the antimicrobial agents tested on the basis of the guidelines provided by the National Committee for Clinical Laboratory Standards (NCCLS) (*24*). Isolates of Salmonella serogroups B and C were further identified to the serotype level, according to the Kauffman and White scheme, by using somatic and flagellar antigens (Denka Seiken Co., Ltd., Tokyo, Japan) and also by conventional methods and the Phoenix System (panel type, NMIC/ID4) (Becton Dickson, Sparks, MD) at hospital A (*25*). Table 1 shows the number of isolates of nontyphoid Salmonella, S. enterica Typhimurium, and S. enterica Choleraesuis, and the ciprofloxacin resistance for the two serotypes of S. enterica isolates recovered from the 11 hospitals.

**Table 1:** Number of ciprofloxacin-resistant nontyphoid Salmonella, S. enterica Typhimurium, and S. enterica Choleraesuis isolates from patients treated at 11 major teaching hospitals in different regions of Taiwan, March 2001–August 2001

Hospital	% (no. ciprofloxacin-resistant isolates/no. total isolates) of ciprofloxacin-resistant isolates of total nontyphoid Salmonella	% (no.ciprofloxacin-resistant isolates/no. total isolates) of ciprofloxacin-resistant isolates, by serotype		
		Typhimurium	Choleraesuis	Others
Northern region	4.3 (7/231)	3.5 (3/85)	7.7 (2/26)	1.7 (2/120)
A	0.0 (0/100)	0.0 (0/40)	0.0 (0/13)	0.0 (0/47)
B	8.2 (4/49)	0.0 (0/19)	28.5 (2/7)	8.7 (2/23)
C	1.7 (1/60)	5.6 (1/18)	0.0 (0/3)	0.0 (0/39)
D	9.1 (2/22)	25.0 (2/8)	0.0 (0/3)	0.0 (0/11)
Central region	3.9 (6/154)	2.4 (2/85)	6.1 (2/33)	5.6 (2/36)
E	5.3 (3/57)	2.8 (1/36)	7.1 (1/7)	7.1 (1/14)
F	7.7 (3/97)	2.0 (1/49)	3.8 (1/26)	4.5 (1/22)
Southern region	2.0 (5/255)	0.0 (0/155)	9.5 (4/42)	1.7 (1/58)
G	0.0 (0/99)	0.0 (0/67)	0.0 (0/11)	0.0 (0/21)
H	2.3 (2/86)	0.0 (0/55)	11.8 (2/17)	0.0 (0/14)
I	4.3 (3/70)	0.0 (0/33)	14.3 (2/14)	4.3 (1/23)
Eastern region				
J	0.0 (0/31)	0.0 (0/22)	0.0 (0/6)	0.0 (0/3)
Total	2.7 (18/671)	1.4 (5/347)	7.5 (8/107)	2.3 (5/217)

A total of 13 isolates of S. enterica Choleraesuis and two isolates of S. enterica Typhimurium recovered from pigs raised in southern (N = 11) and central (N = 4) Taiwan from 1997 to 2002 were also collected for study. Of the 15 isolates, 2 each were collected in 1997, 1999, and 2001 and 3 each in 1998, 2000, and 2002. These isolates were collected from various visceral organs (lungs, liver, or spleen) or from stool specimens of pigs that died of septicemia. All the isolates were stored at –70°C in trypticase soy broth (Difco Laboratories, Detroit, MI) supplemented with 15% glycerol before being further tested. S. enterica Choleraesuis ATCC 13312 and S. enterica Typhimurium ATCC 14028 were used as control strains.

### Prevalence of Resistance at Hospital A

To determine the prevalence of antimicrobial resistance among nontyphoid Salmonella, we analyzed the disk diffusion susceptibility results of these organisms to ampicillin, cefotaxime/ceftriaxone, chloramphenicol, trimethoprim-sulfamethoxazole, and ciprofloxacin recovered from 1996 to 2001 at hospital A. Isolates of Salmonella serogroups B and C resistant to ciprofloxacin (by the disk diffusion method) were further identified to the serotype level by the methods mentioned above.

### Antimicrobial Susceptibility Testing

Of the 671 isolates collected from 11 medical centers in 2001, 37 ciprofloxacin-resistant (by the disk diffusion method) S. enterica Typhimurium (N = 26) and S. enterica Choleraesuis (N = 11) isolates and 10 randomly selected ciprofloxacin-susceptible (by the disk diffusion method) isolates of these two serotypes were tested for susceptibility to six fluoroquinolones (ciprofloxacin, levofloxacin, moxifloxacin, trovafloxacin, gatifloxacin, and garenoxacin) to determine their MICs by using the agar dilution method according to the guidelines established by NCCLS (*26*). The 37 ciprofloxacin-resistant isolates included 13 isolates from the 2001 SMART program (5 S. enterica Typhimurium isolates and 8 S. enterica Choleraesuis isolates) and 24 recovered from 1996 to 2000 at hospital A (21 S. enterica Typhimurium isolates and 3 S. enterica Choleraesuis isolates). These ciprofloxacin-resistant isolates were recovered from 29 patients. Six patients (patients 9, 10, 11, 15, 29, and 32) had isolates that were recovered after >7 days from various clinical specimens. The patients’ ages ranged from <1 year to 84 years (mean 31 years); those <2 years of age were predominant (47%) among patients with S. enterica serotype Typhimurium isolations. None of the patients with S. enterica Choleraesuis bacteremia were <16 years. Among the 37 human isolates of ciprofloxacin-resistant nontyphoid Salmonella isolates, 13 (3 of S. enterica Typhimurium and 10 of S. enterica Choleraesuis) were recovered from blood specimens of 12 patients with bloodstream infections. The rest of the isolates were recovered from stool or urine specimens.

 Dilution susceptibilities to the aforementioned fluoroquinolones were also performed for the 15 isolates from pig herds, according to the NCCLS guidelines (*26*).

### PCR Amplification and DNA Sequencing of gyrA, gyrB, parC, parE, and acrR

 The sequences of the primers for the polyermase chain reaction (PCR) amplification of gryA, gyrB, parC, parE, and acrR have been previously described (*27*–*30*). The preparation of the template DNA and the determination of sequences of each gene followed the procedures described previously (*27*–*29*). The sequences of the quinolone resistance-determining regions (QRDRs) were determined to be between amino acids 54 and 171 of gyrA, 397 and 520 of gyrB, 12 and 130 of parC, and 421 and 524 of parE.

### Ciprofloxacin Accumulation

The accumulation of ciprofloxacin, with or without 100 μM carbonyl cyanide m-chlorophenylhydrazone, was determined for two ciprofloxacin-resistant strains and one ciprofloxacin-susceptible S. enterica Typhimurium (ciprofloxacin MIC = 0.06 μg/mL) as described previously (*28*,*29*). These experiments were performed twice to ensure reproducibility.

### Molecular Typing

 Genotyping of the human ciprofloxacin-resistant S. enterica Typhimurium (N = 26) and Choleraesuis (N = 11) isolates, the 10 human ciprofloxacin-susceptible isolates of the two serotypes, and the 15 isolates from pigs was determined by the pulsotypes generated by pulsed-field gel electrophoresis (PFGE). The DNA extraction and purification were also carried out as described previously (*32*). The DNA was digested by the restriction enzymes SpeI, XbaI, and BlnI (*9*,*16*,*23*,*32*), and the restriction fragments were separated in a CHEF-DRIII unit (Bio-Rad, Hercules, CA). Interpretation of the PFGE profiles followed the description by Tenover et al. (*33*). Isolates belonging to the similar pulsotypes (within six band differences) by each of the three restriction enzymes were defined as the same genotypes (closely related clusters). Isolates with identical pulsosubtypes (no band differences) by the three restriction enzymes were defined as the same genosubtypes (clones).

##  Results

### Nationwide Resistance in 2001

 The rates of ciprofloxacin resistance among isolates of nontyphoid Salmonella, S. enterica Typhimurium, and S. enterica Choleraesuis from the 11 hospitals, stratified by region of Taiwan, is shown in Table 1. Overall, ciprofloxacin resistance was found in 2.7% (18/671) of all nontyphoid Salmonella isolates from humans, 1.4% in S. enterica Typhimurium and 7.5% in S. enterica Choleraesuis nationwide (Table 1). Among S. enterica Choleraesuis isolates, the highest rate of ciprofloxacin resistance was found in hospital B (28.5%) and southern region of Taiwan (9.5%). Among S. enterica Typhimurium isolates, the highest rate of ciprofloxacin resistance was found in hospital D (25.0%) and in the northern region of Taiwan (3.5%). Nontyphoid Salmonella isolates recovered from patients in eastern region of Taiwan were all susceptible to ciprofloxacin. Rates of resistance to ampicillin and chloramphenicol were higher in eastern Taiwan than those from other regions of Taiwan. Resistance to cefotaxime (three hospitals tested ceftriaxone instead of cefotaxime) among all nontyphoid Salmonella isolates was low (<1%). However, 6% and 4% of S. enterica Choleraesuis isolates recovered from central and southern Taiwan, respectively, were resistant to cefotaxime (ceftriaxone).

### Prevalence of Ciprofloxacin Resistance at Hospital A

The annual number of nontyphoid Salmonella isolates (Salmonella group B and Salmonella group C) ranged from 294 in 1996 (182 and 46, respectively) to 90 in 2001 (76 and 8, respectively). Overall, the rate of ciprofloxacin resistance among nontyphoid Salmonella isolates was 2.1%. For Salmonella group B isolates, the rates of ciprofloxacin resistance were high (6% to 9%) during 1996 and 1997, reached a trough in 1999 (3%), and increased gradually in the following 2 years (4% in 2000 to 5% in 2001). Annual rates of resistance to ciprofloxacin among Salmonella group C isolates fluctuated during the same 6-year period (data not shown). In 1996, 1998, and 2001, none of the isolates were resistant to ciprofloxacin, and the highest rate of ciprofloxacin resistance was found in 2000 (13%).

 The annual rates of resistance to ampicillin, cefotaxime, chloramphenicol, and trimethoprim-sulfamethoxazole among all nontyphoid Salmonella isolates, Salmonella group B, and Salmonella group C at hospital A from 1996 to 2001 were evaluated. Overall, the prevalence of resistance to cefotaxime among these isolates was low (0% to 4%). Rates of resistance to ampicillin, chloramphenicol, and trimethoprim-sulfamethoxazole among all nontyphoid Salmonella isolates declined gradually from 1996 (64%, 64%, and 42%, respectively) to 2000 (47%, 52%, and 34%, respectively). In 2001, however, rates of resistance to ampicillin (73%) and chloramphenicol (76%) increased but that of trimethoprim-sulfamethoxazole (28%) continued to decrease. A similar scenario was found among Salmonella group B isolates. For Salmonella group C isolates, rates of resistance to these agents also fluctuated during the study period.

### Antimicrobial Susceptibilities of Human Ciprofloxacin-resistant Isolates

All of the human ciprofloxacin-resistant isolates were highly resistant to ampicillin (MIC >128 μg/mL), chloramphenicol (MIC >128 μg/mL), and trimethoprim-sulfamethoxazole (>128 μg/mL) but susceptible to cefotaxime (MIC 0.06–8 μg/mL). These ciprofloxacin-resistant isolates from humans all exhibited high-levels of resistance to ciprofloxacin (MIC 8 to 64 μg/mL), levofloxacin (MIC 32–64 μg/mL), moxifloxacin (MIC 32–28 μg/mL), gatifloxacin (MIC 16–32 μg/mL), garenoxacin (MIC 16–64 μg/mL), and trovafloxacin (MIC 8–64 μg/mL).

### Fluoroquinolone Susceptibilities among Swine S. enterica Choleraesuis Isolates

 All 15 isolates of S. enterica Typhimurium and Choleraesuis from pigs had reduced susceptibility to ciprofloxacin (MIC >0.125 μg/mL). Eight of the 15 isolates (53%) were susceptible or intermediate to ciprofloxacin according to the NCCLS breakpoint recommendation (MIC <2 μg/mL). Seven (47%) isolates had high ciprofloxacin MICs (MIC >64 μg/mL); these seven isolates were also highly resistant to five other newer fluoroquinolones: levofloxacin (MIC 32–64 μg/mL), moxifloxacin (MIC 32–128 μg/mL), trovafloxacin (MIC 64 μg/mL), gatifloxacin (MIC 16–32 μg/mL), and garenoxacin (MIC 32–64 μg/mL).

### Nucleotide Sequence Analysis

 Of human ciprofloxacin-resistant S. enterica Typhimurium isolates, all were associated with two-base substitutions in the QRDR of gyrA at codon 83 (Ser83Phe) (TCC→TTC) and 87 (Asp87Gly) (GAC→GGC), and either Ser80Arg or Glu84Lys in the QRDR of the parC gene (Table 2). One base substitution in the QRDR of gyrA (Ser83Tyr or Ser83Phe) was found in ciprofloxacin-susceptible isolates. One isolate had a mutation in the QRDR of the gyrB gene, but none had mutations in the QRDR of the parE gene. None of the S. enterica serotype Typhimurium isolates, including ciprofloxacin-susceptible or -resistant isolates, had mutations in the acrR genes.

**Table 2:** Characteristics of Salmonella enterica serotype Typhimurium isolates from humansa and pigs,b Taiwan

Ciprofloxacin susceptibility (N)	MIC (μg/mL) (N)		Mutation at												Genotype: genosubtype
			gyrA gene				parC gene					acrR gene			
			Ser83Phe Asp87Gly (N)	Ser83Phe Asp87Asn (N)	Ser83Ph (N)e		Ser83Tyr (N)	Ser80Arg (N)		Glu84Lys (N)		Gln78Stp (N)		Arg107Cys (N)	
Humans															
Resistant (26)		16-64	24	2	0	0		24	2		0		0		I (26), Ia (4), Ic (5), Id (6), Ie (1), If (1), Ig (2), Ih (1), Ii (1) Ij (1), Ik (2), Il (1)
Susceptible (5)		0.03-0.25	0	0	1	2		0	0		0		0		IIa (1), IIb (1), III (1), IV (1), V (1)
Pigs															
Resistant (2)		128	2	0	0	0		2	0		0		0		I (2): Ic (1), Im (1)

aN = 31. bN = 2.

 Of human ciprofloxacin-resistant S. enterica serotype Choleraesuis, all were associated with two-base substitutions in the QRDR of the gyrA gene at codon 83 (Ser83Phe) and 87 (Asp87Asn), Ser80Ile in the QRDR of the parC gene, and Gln78Stp in the QRDR of the acrR gene (Table 3). None of these isolates had mutations in the QRDR of the gyrB or parE genes. One base substitution in the QRDR of the gyrA (Asp87Asn) was found in the ciprofloxacin-susceptible isolates.

**Table 3:** Characteristics of Salmonella. enterica serotype Choleraesuis isolates from humansaand pigsb in Taiwan

Ciprofloxacin susceptibility (μg/mol)	MIC	Mutation at								Genotype: genosubtype (N)
		gyrA gene					parC gene	acrR gene		
		Ser83Phe Asp87Asn (N)	Ser83Phe (N)	Asp87Asn (N)	Ser83Tyr (N)	Asp87Gyr (N)	Ser80Ile (N)	Gln78Stp (N)	Arg107Cys (N)	Ser83Phe (N)
Humans										
Resistant (11)	16–64	11	0	0	0	0	0	0	0	A (1); B (10): B1 (2), B2 (2), B3 (2), B4 (1), B5 (2), B6 (1)
										
Susceptible (5)	0.03–0.25	0	0	1	0	0	0	0	0	C (1), D (1), E (1), F (1), G (1)
Pigs										
Resistant (5)	64	5	5	0	0	0	5	5	0	0 B (5): B2 (1), B5 (1), B8 (1), B9 (2)
Susceptible or intermediate (8)	0.5–2	0	1	1	2	4	0	0	0	B (2): B7 (1), B10 (1); H (1), I (1), J (3), K (1)

aN = 16. bN = 13.

Of pig herd ciprofloxacin-resistant S. enterica serotype Typhimurium isolates (N = 2), both had a mutation in the QRDR of the gyrA and parC genes, respectively (Table 2). None of these isolates had mutations in the QRDR of the gyrB, parE, or arcR genes. Among pig herd ciprofloxacin-resistant S. enterica serotype Choleraesuis isolates, all had two mutations in the QRDR of gyrA (Ser83Phe plus Asp87Asn or Asp87Gly) and one mutation in parC (Ser80Ile) and arcR (Gln78Stp) (Table 3).

### PFGE Analysis and Genotypes

**Figure 1:**
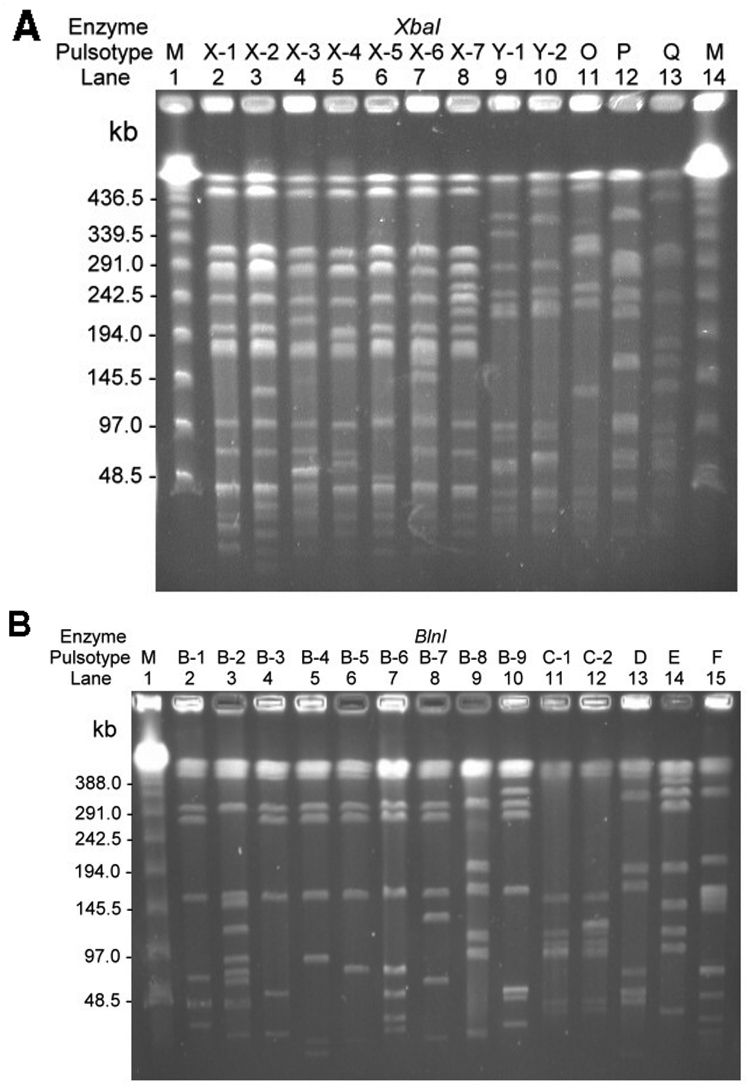
Pulsotypes and? pulsosubtypes of S. enterica serotype Typhimurium from humans and pigs obtained by pulsed-field gel electrophoresis (PFGE) after digestion with XbaI (A) and BlnI. (B). Lanes M, molecular size marker. See for designations. See Table 2 and Table 3 for the designation of isolates for each indicated pulsotype or pulsosubtype.

**Figure 2:**
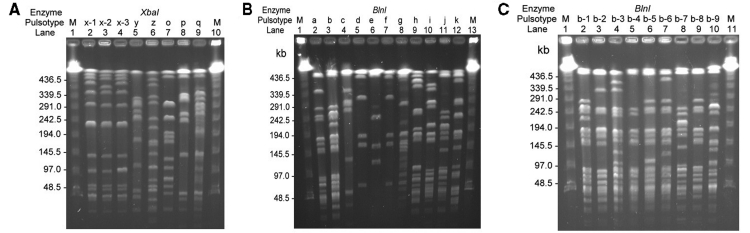
Pulsotypes and pulsosubtypes of Salmonella enterica serotype Choleraesuis from humans and pigs obtained by pulsed-field gel electrophoresis (PFGE) after digestion with XbaI (A) and BlnI (B and C). Lanes M, molecular size marker. See for designations. See Tables 2 and 3 for the designation of isolates for each indicated pulsotype or pulsosubtype.

All of the ciprofloxacin-resistant S. enterica serotype Typhimurium isolates from humans had the same pulsotype (pulsotype S) when the SpeI restriction enzyme was used. Figure 1 and Figure 2 illustrate the pulsotypes and pulsosubtypes of S. enterica Typhimurium (Figure 1A and 1B) and S. enterica Choleraesuis (Figure 2A, 2B, and 2C) isolates by XbaI and BlnI. Using XbaI and BlnI, we observed six and eight pulsosubtypes, respectively, for S. enterica Typhimurium isolates. Among S. enterica Choleraesuis isolates, one pulsotype (x) and one pulsosubtype (x-1) were observed when the XbaI restriction enzyme was used, and two pulsotypes (a and b) with six pulsosubtypes (b-1 to b-6) were observed when the BlnI restriction enzyme was used. Using the three restriction enzymes, we found that all ciprofloxacin-susceptible isolates of S. enterica Typhimurium and Choleraesuis had different genosubtypes (clones).

Among human ciprofloxacin-resistant S. enterica Typhimurium isolates, all were closely related (genotype I) and belonged to 11 genosubtypes (genosubtypes Ia to Ik). Among the 11 genosubtypes, Ia (4 isolates), Ic (5 isolates), and Id (6 isolates) predominated. The five ciprofloxacin-susceptible isolates belonged to four genotypes (II–V) (Table 2). None of the genotypes among the S. enterica serotype Typhimurium isolates studied were identical to those of DT104.

Of human S. enterica Choleraesuis isolates, 91% (10 of the 11 isolates) belonged to genotype B, which was different from those of the five ciprofloxacin-susceptible isolates from humans (genotypes C to G). None of the six genosubtypes (B1 to B6) of the genotype B isolates was predominant. Two isolates collected within 7 days of one another from patient 4 had identical genosubtypes (B2), but those from patient 7 had differing genosubtypes (B4 and B5) (Table 3).

Seven (54%) of the 13 swine S. enterica Choleraesuis isolates belonged to genotype B (Table 3). Among the six genosubtypes of genotype B, two genosubtypes (B2 and B5) were also found in human isolates. Two swine isolates that showed descreased susceptibility to ciprofloxacin (MICs, 0.5 μg/mL and 2 μg/mL, respectively) also belonged to genotype B (genosubtypes B7 and B10, respectively).

### Evidence for Active Efflux

**Figure 3:**
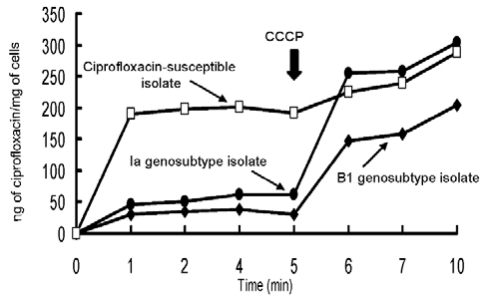
Accumulation of ciprofloxacin by the two ciprofloxacin-resistant isolates of genosubtype Ia of Salmonella enterica serotype Typhimurium and genosubtype B1 of S. enterica serotype Choleraesuis and one clinical isolate of S. enterica serotype Typhimurium (ciprofloxacin MIC = 0.06 μg/mL). Carbonyl cyanide m-chlorophenylhydrazone (CCCP) (100 μM) was added at the time indicated by the arrow.

 Ciprofloxacin uptake appeared to be remarkably low in the two ciprofloxacin-resistant genotypes (genosubtypes Ia and B1) (Figure 3). A rapid increase in cell-associated ciprofloxacin uptake among isolates belonging to the genosubtypes was evident after addition of carbonyl cyanide m-chlorophenylhydrazone (CCCP), a proton motive force uncoupler.

## Discussion

This report describes the rates of antimicrobial resistance among nontyphoid Salmonella isolates in a university hospital during a 6-year period. Furthermore, it characterizes the nationwide dissemination of ciprofloxacin-resistant strains of S. enterica Typhimurium and Choleraesuis. Our observations in Taiwan suggest five important facets: First, ciprofloxacin resistance among our nontyphoid Salmonella from humans was high (2.7%), particularly among human S. enterica Choleraesuis isolates. Furthermore, a previous study found a remarkably high incidence (57%, 12 of 21 isolates) of S. enterica Choleraesuis at two major teaching hospitals in northern Taiwan in the second to third quarters of 2001 (*14*). This observation contrasts greatly with our findings (7.7%, 2 of 25 isolates, from four hospitals in northern Taiwan, or 7.5% nationwide from March 2001 to August 2001) (*14*).

Second, nearly all ciprofloxacin-resistant S. enterica Choleraesuis isolates from humans and pigs collected in 1999–2002 were closely related to one another (genotype B) and differed from those found in pigs in 1997–1998 (these isolates had highly diverse genotypes). These findings suggest that nationwide dissemination of S. enterica Choleraesuis isolates from pigs to humans occurred from 1999 to 2002. Two isolates (AC-6 and AC-10) of S. enterica Choleraesuis from pigs had reduced susceptibility to ciprofloxacin (MICs, 0.5 μg/mL and 2 μg/mL, respectively). They had an identical genotype (genotype B) to that of most of the epidemic strains found in humans and pigs. These strains had high-level ciprofloxacin resistance (MIC 16 to 64 μg/mL). This indicates that the swine isolates with reduced susceptibility to ciprofloxacin might be an ancestor (a unique clone line) of the isolates that are highly resistant to ciprofloxacin and which have spread among herbs and humans nationwide (*23*,*27*).

Third, the S. enterica Typhimurium strains (genotype I) with high-level fluoroquinolone resistance have been widely disseminated in humans in Taiwan since 1996. Strains belonging to genotype I and the other genotypes found in this study were domestically acquired and were not related to the clones of DT104, which were already disseminated throughout Europe and the United States (*15*,*23*). In 1998, one isolate exhibiting genotype I (genosubtype Ic) was isolated from a pig from southern Taiwan. Further studies on S. enterica Typhimurium isolates from animals should be conducted to identify the primary source of the epidemic genotype strains.

Fourth, an increasing prevalence of resistance to ampicillin and chloramphenicol over time was observed in human S. enterica Typhimurium isolates at hospital A. The spread of third-generation cephalosporin-resistant isolates harboring plasmid-mediated CMY-2 like cephalosporinase among S. enterica Typhimurium isolates has been previously reported in Taiwan (*6*). Although all of the highly ciprofloxacin-resistant isolates in our study were susceptible to cefotaxime, according to NCCLS guidelines (*24*), five isolates from four patients with high cefotaxime MICs (MIC 4–8 μg/mL) is noteworthy. The emergence of decreased susceptibility to cefotaxime, along with the preexisting ciprofloxacin resistance among nontyphoid Salmonella isolates, particularly those causing bloodstream infection, makes antimicrobial therapy more complicated.

Finally, rates of resistance varied geographically; higher rates of resistance to ampicillin and chloramphenicol were found in the eastern region of Taiwan. However, none of the nontyphoid Salmonella isolates collected in the eastern region of Taiwan was resistant to ciprofloxacin.

S. enterica Typhimurium and S. enterica Choleraesuis isolates with high levels of resistance to ampicillin, chloramphenicol, trimethoprim-sulfamethoxazole, ciprofloxacin, and other newer fluoroquinolones were rarely previously reported (*2*,*19*,*28*,*30*,*34*). In most gram-negative bacteria, including Salmonella, a high-level of fluoroquinolone resistance is related to the presence of multiple mutations in the QRDRs of the genes, particularly in the gyrA and parC genes (*8*,*13*,*14*,*19*,*27*,*28*,*35*).

Additional resistance mechanisms, such as decreased cell envelope permeability (loss of outer membrane porins or alterations of the lipopolysaccharide), decreased cellular accumulation of quinolones involving the major multidrug active efflux pump (AcrAB), or the presence of integrons, can also be responsible for fluoroquinolones resistance and resistance to a wide range of antimicrobial agents (*31*,*35*–*39*). Mutations in the acrR (regulator/repressor) gene are partly responsible for fluoroquinolone resistance in Escherichia coli (*29*). In our study, the two major genotypes (genotypes I and B) of ciprofloxacin-resistant isolates had both mutations in the gyrA (at least two mutations) and parC (at least one mutation) genes. The addition of CCCP, resulted in an increase in cell-associated ciprofloxacin uptake. This indicated that an active efflux contributed to fluoroquinolone resistance (*28*–*31*). In our study, acrR mutations were found in ciprofloxacin-resistant S. enterica Choleraesuis, but not in Typhimurium, isolates. This finding is consistent with that of previous reports (*30*). Further studies are warranted to add clarification to the complexity of the mechanisms of high-level resistance among S. enterica Typhimurium and Choleraesuis isolates.

PFGE analysis using restriction enzyme XbaI is a well-established method for epidemiologic typing of the Salmonella species (*9*,*16*,*23*,*32*). However, PFGE patterns by XbaI for most ciprofloxacin-resistant isolates investigated, including S. enterica Choleraesuis isolates from human and animal origins, were indistinguishable. This scenario was also found in PFGE patterns for human S. enterica Typhimurium isolates. When BlnI was added, the discriminatory power of pulsotyping improved among these ciprofloxacin-resistant isolates. Genotyping by using pulsotypes generated by XbaI and BlnI clearly demonstrated that several ciprofloxacin-resistant clones (particularly, genosubtypes Ia, Ic, and Id of S. enterica Typhimurium) had disseminated to humans in Taiwan. Furthermore, some genosubtypes of ciprofloxacin-resistant S. enterica Choleraesuis were found only in humans, and some were found only in pigs; however, two clones (genosubtypes B2 and B5) were found in both humans and pigs.

Research studies have provided evidence that antimicrobial agents used in agriculture and closely related agents used in human medicine have been exerting selective pressure on their target bacteria, particularly Salmonella, Campylobacter, and Escherichia coli (*1*,*20*,*40*). In Taiwan, quinolones (e.g., enrofloxacin) have been used in animals and humans (from nalidixic acid to the latest fluoroquinolone moxifloxacin) for >30 years. A governmental survey among farmers and feed mill operators in 1999 indicated that 40% of farmers and 50% of feed mill operators used quinolone agents (particularly enrofloxacin) on their flocks or herds of pigs for growth promotion or therapeutic purposes (*41*). Previous investigations demonstrated that >90% of Campylobacter species and 6% of E. coli from chickens were resistant to ciprofloxacin (*41*,*42*). When the selective pressure of quinolones persisted, isolates, or some clones with reduced susceptibility (a single gyrA mutation) to quinolones, might develop full resistance (two gyrA mutations or multiple mutations in the QRDRs other genes) in animals or humans and could probably jump from animals to humans (*14*,*27*). Our observations and findings from Chiu et al. indicate that outbreak-associated human Salmonella strains with high-level ciprofloxacin resistance might have emerged several years ago, similar to strains with antibiotypes of reduced susceptibility, but with identical genotypes, in humans or animals (*14*).

 In conclusion, our investigation documented that S. enterica Typhimurium and S. enterica Choloraesuis isolates, which are highly fluoroquinolone-resistant and multidrug-resistant, have become widespread pathogens in Taiwan. The recent occurrence of ciprofloxacin resistance among Salmonella in animals, and its nationwide spread, is of particular concern. The remaining therapeutic options available to veterinarians and physicians for treatment of extraintestinal salmonellosis and other invasive infections include only third-generation cephalosporins. However, ciprofloxacin-resistant isolates, with reduced susceptibility to cefotaxime, have already emerged in Taiwan. Restricted use of quinolones in animal husbandry and active surveillance of quinolone resistance among Salmonella are crucial mitigation efforts to reduce selection and clonal spread of quinolone-resistant Salmonella.
